# Actinomycosis of Gallbladder in Cholecystectomy Specimen: A Case Report

**DOI:** 10.31729/jnma.6709

**Published:** 2021-11-30

**Authors:** Brijesh Shrestha, Manisha Regmi, Prabesh Adhikari

**Affiliations:** 1 Department of Pathology, National Medical College and Teaching Hospital, Birgunj, Nepal

**Keywords:** *actinomycosis*, *case report*, *cholelithiasis*, *gallbladder*, *Nepal*

## Abstract

Actinomyces are a part of the normal flora of the cervicofacial region, gastrointestinal tract, and urogenital tract, but can cause infections when the normal mucosal barrier is lost. Herein, we report a rare case of actinomycosis of the gallbladder in a 60-years-old-female. The patient presented with right hypochondrium pain since three months; ultrasonography showed cholelithiasis with thick oedematous wall. An open cholecystectomy was carried out. Histological examination revealed an inflamed gallbladder with colonies of radiating filamentous structures having numerous sulphur granules which on gram staining showed filamentous gram-positive rods. The diagnosis of Actinomycosis of gallbladder was made. After cholecystectomy, prolonged antimicrobial therapy is recommended for patient with actinomycosis to prevent recurrence and even mortality.

## INTRODUCTION

Actinomycosis is a chronic suppurative disease caused by Actinomyces, a gram positive, anaerobic filamentous bacterium. It is a part of the normal microflora in cervicofacial region and gastrointestinal tract. However, the disease occurs when normal mucosal barrier is lost due to trauma, surgery or infection. It usually spreads invading tissue planes of the body forming abscess that finally leads to the formation of draining sinuses. Actinomyces israelii is the most common species.^1,2^ Actinomycosis of the gallbladder is extremely rare; herein we report a case of actinomycosis of the gallbladder which could probably be the first case published in Nepal.

## CASE REPORT

A 60-years-old-female presented with dull aching pain at the right hypochondrium since three months with no history of abdominal surgery and no significant medical history. On physical examination, there was tenderness in the right hypochondrium. Ultrasonography of the abdomen revealed cholelithiasis with thick gallbladder wall. Laboratory studies revealed normal liver function tests, amylase and serum electrolytes. Human immunodeficiency virus, Hepatitis B, and Hepatitis C serology were negative. The pre-operative diagnosis was symptomatic cholelithiasis.

The patient was admitted and an open cholecystectomy was performed. Gall bladder was received for histopathological examination.

**Figure 1:**
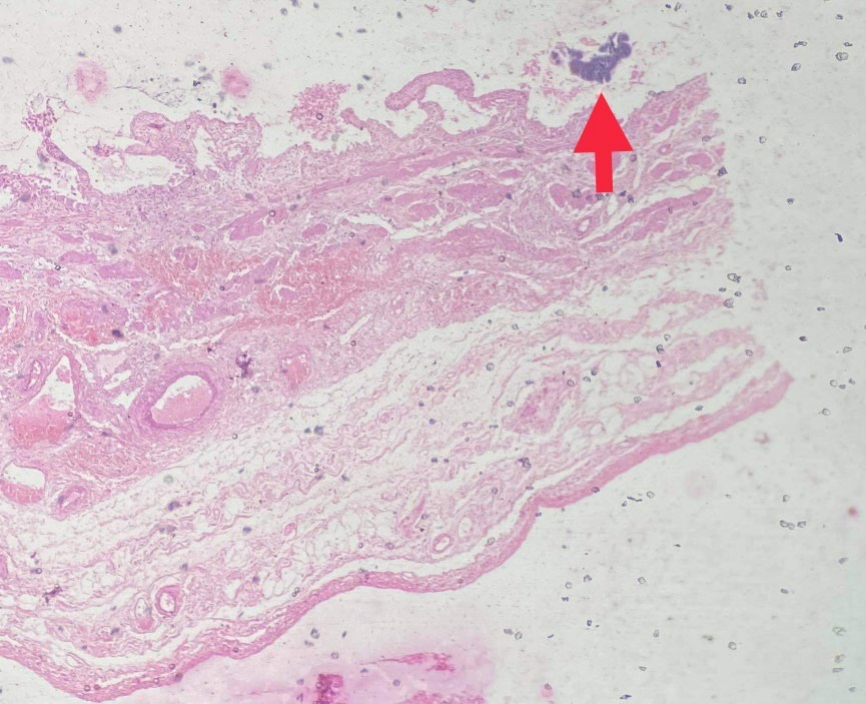
Colonies of Actinomyces shown by red arrow (Hematoxylin and Eosin stain, 4X).

**Figure 2:**
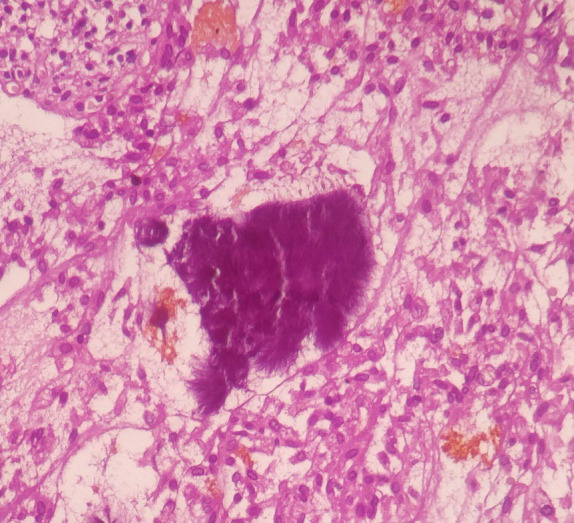
Characteristic 'sulphur granules' composed of basophilic radiating filaments of *Actinomyces* (Hematoxylin and Eosin stain, 20X).

**Figure 3:**
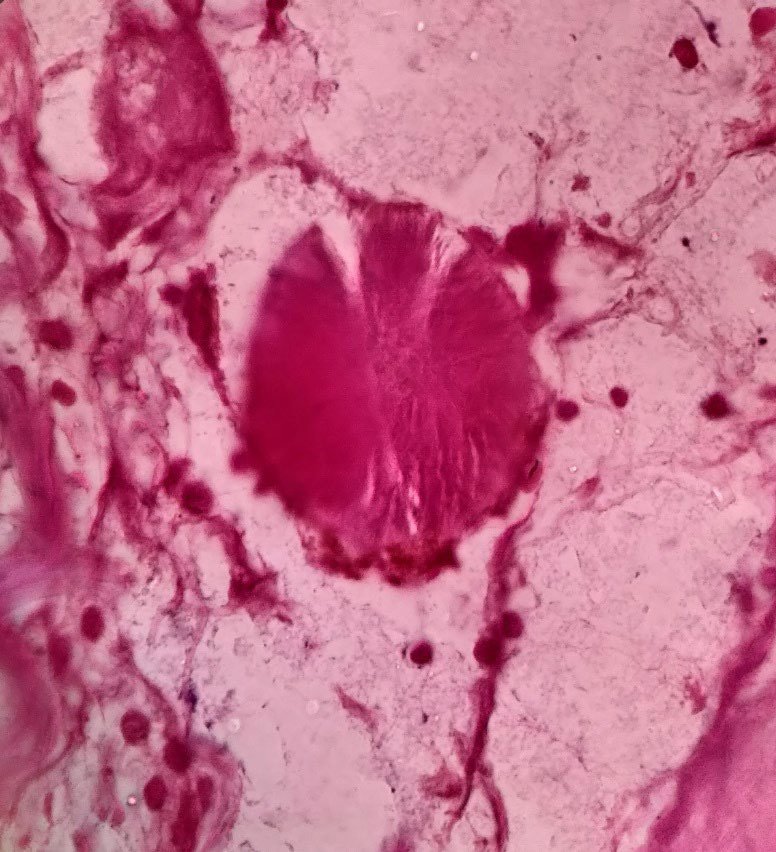
Filamentous bacteria stain positive with Gram stain (Gram stain, 20X).

Gross examination revealed the gallbladder measuring 9.5x2.5x0.8 cm. The mucosa was bile stained and gall bladder wall was thickened measuring 0.8 to 1 cm. On cut section, a single grayish white calculi measuring 1x1cm was seen. Microscopic examination revealed focally denuded mucosal epithelium with many colonies of radiating filamentous structures having numerous sulphur granules (Figure 1,2) which on gram staining showed filamentous gram-positive rods (Figure 3).

Follow-up was advised to the patient after two weeks but the patient did not present on the follow-up. So, we lost contact with the patient and the outcome was unable to assess.

## DISCUSSION

Actinomycosis is a chronic infection caused by Actinomyces species which is an anaerobic grampositive filamentous bacterium. There are more than 30 species under the genus Actinomyces, the most common species are Actinomyces israelii, A. gerenseriae and A. naeslundii.^1^ The bacteria are commensal of the oral cavity, gastrointestinal tract and urogenital tract and have low pathogenicity, but any breech in tissue barriers by trauma, surgery or infection can facilitate the organism to penetrate and cause disease.^2-4^ Actinomycosis is also associated with other risk factors like immunosuppression, neoplasia, diabetes and intrauterine contraceptive devices (IUDs).^3^ It is endemic globally, prevalent more commonly in developing countries with poor socioeconomic conditions like Nepal.

Actinomycosis most commonly presents in the cervicofacial infection followed by abdomino-pelvic region. Gallbladder actinomycosis is a very rare finding and there are very few cases reported in literature. Most cases are associated with gallstone or other underlying diseases.^3^ Mir, et al. reviewed 16 cases with gallbladder and bile duct actinomycosis. They found that 10 cases had the stones in gallbladder or bile duct, two cases had diabetes mellitus and eight cases were associated with other diseases including liver tumor, liver abscess, gallbladder carcinoma, amyloidosis, myocardial infarction, rheumatoid arthritis, glomerulonephritis and heart failure. Only seven cases were not affected by any underlying disease.^5^ In our case, the patient had gallbladder stone but no other underlying conditions. The stone could have predisposed to actinomycosis following bacterial reflux from the duodenum into the inflamed gallbladder. Most studies show the mean age at presentation to be 65 years and is more common in females. It is consistent with our case who was a 60 years old female.

It is difficult to diagnose gallbladder actinomycosis clinically, it may present as acute or chronic cholecystitis and often mimics carcinoma radiographically. Histolopathological diagnosis of actinomycosis is based on the demonstration of "sulfur granules" a dense aggregate of filamentous Actinomyces associated with suppurative inflammation. However, sulfur granules can occasionally be found in other infections such as Nocardia brasiliensis, Streptomyces madurae, and Staphylococcus aureus. Although sulfur granules are seen only in 50% of patients with actinomycosis, the micro-organism presenting as filamentous gram positive bacteria are pathognomic for diagnosing actinomycosis.^1, 2^

After cholecystectomy, prolonged antimicrobial therapy is recommended for patient with actinomycosis to prevent recurrence. The treatment of choice is high dose intravenous penicillin followed by oral penicillin alone or combined with ß-lactamase inhibitor (i.e. clavulanate, tazobactum) to cover both aerobic and anaerobic bacteria.^6^

Actinomycosis of gallbladder is a very rare condition and poses a great diagnostic challenge because of its non-specific symptoms and wide variety of clinical presentation as it tends to mimic different diseases such as symptomatic cholelithiasis and gallbladder carcinoma. The diagnosis is usually made by histological examination of the cholecystectomy specimen. The treatment needs to be long term antibiotics to avoid recurrence and even mortality.
